# Predictors of In-Hospital Mortality From Infective Endocarditis in a Resource-Limited, War-Affected Tertiary Center

**DOI:** 10.31083/RCM49339

**Published:** 2026-04-22

**Authors:** Mohammed Abdul-Malik Ghalib, Mohammed Al-Kebsi, Taha Al-Maimoony, Abdulhafeedh Al-Habeet

**Affiliations:** ^1^Department of Cardiology, Faculty of Medicine and Health Sciences, Sana'a University, Sana'a, Yemen; ^2^Department of Epidemiology and Biostatistics, Faculty of Medical Sciences, Al-Razi University, Sana'a, Yemen

**Keywords:** infective endocarditis, in-hospital mortality, resource-limited settings, armed conflicts, septic shock

## Abstract

**Background:**

Infective endocarditis (IE) carries a high in-hospital mortality, particularly in resource-limited and war-affected settings. However, data on short-term mortality predictors in such environments remain limited. Therefore, this study aimed to identify independent predictors of in-hospital mortality among patients with IE treated at a tertiary referral center in Yemen.

**Methods:**

A prospective cohort study was conducted between October 2023 and August 2025 at the largest tertiary referral center in Yemen. A total of 60 consecutive patients with IE (46 with definite IE and 14 with possible IE) were included, diagnosed according to the modified Duke criteria. Candidate predictors were screened using univariable analyses. Given the limited number of outcome events, the least absolute shrinkage and selection operator (LASSO) regression was applied for variable selection, followed by Firth's penalized logistic regression to obtain bias-reduced estimates.

**Results:**

A total of 17 patients died during hospitalization, yielding an in-hospital mortality rate of 28.3%. Baseline demographic characteristics, microbiological findings, and most echocardiographic parameters were not independently associated with mortality. However, in-hospital complications showed strong associations with death. In the final penalized multivariable model, septic shock (adjusted odds ratio (AOR) 14.441; 95% confidence interval (CI): 2.242–176.650; *p* = 0.004) and acute kidney injury (AKI) (AOR 5.286; 95% CI: 1.226–26.440; *p* = 0.0264) emerged as the most robust independent predictors of in-hospital mortality, whereas uncontrolled infection did not retain statistical significance.

**Conclusions:**

In this war-affected and resource-limited setting, in-hospital mortality from IE was substantial and driven primarily by severe systemic complications. Early recognition and aggressive management of septic shock and AKI may improve short-term outcomes in similar low-resource environments.

## 1. Introduction

Infective endocarditis (IE) remains a life-threatening condition characterized by infection of the endocardial surface of the heart, often leading to severe complications including acute heart failure (AHF), systemic embolization, and uncontrolled infection [1, 2, 3]. Globally, the reported in-hospital mortality rates for IE vary widely, ranging from 15% to 50% [4, 5, 6, 7, 8, 9, 10, 11]. This likely reflects heterogeneity in patient populations, pathogen profiles, and healthcare infrastructure.

The diagnostic approach to IE has evolved substantially in recent years. The 2023 European Society of Cardiology (ESC) guidelines for the management of IE now integrate advanced imaging modalities into the diagnostic algorithm, including ^18^F-fluorodeoxyglucose positron emission tomography/computed tomography and cardiac computed tomography angiography [12]. These modalities have improved the detection of perivalvular complications and embolic events, particularly in patients with prosthetic valves or intracardiac devices. Concurrently, the 2023 Duke-International Society for Cardiovascular Infectious Diseases (ISCVID) criteria have expanded the diagnostic framework by incorporating these advanced imaging findings, newer microbiological techniques, and updated histopathological definitions, thereby improving diagnostic sensitivity and specificity [13].

Previous studies have consistently identified several independent predictors of in-hospital mortality in patients with IE, including older age [5, 14, 15], prior stroke [10], septic shock [11, 14, 16], heart failure (HF) [5, 8, 11, 16], acute kidney injury (AKI) [5], cerebrovascular complications [5, 17], embolic complications [18, 19], prosthetic valve endocarditis (PVE) [19], and the absence of early surgery in patients with established surgical indications [5, 16]. However, most of this evidence originates from high-income countries with timely access to advanced diagnostics, multidisciplinary endocarditis teams, and early surgical intervention. These findings may not be directly applicable to resource-limited and war-affected settings, where delayed presentation, limited microbiological yield, constrained intensive care capacity, and restricted access to cardiac surgery are common. In such environments, patients often present with advanced disease and severe systemic complications, potentially modifying the relative impact of traditional prognostic factors on in-hospital mortality.

Yemen has been affected by a prolonged armed conflict since late 2014, with a marked escalation following the onset of regional military intervention in early 2015. Over more than a decade, this conflict has led to widespread destruction of healthcare infrastructure, shortages of medical supplies and trained personnel, and major disruptions in referral networks and continuity of care. Access to advanced diagnostic modalities, intensive care services, and cardiac surgery has become severely constrained, while delays in seeking medical attention have become common due to insecurity and socioeconomic collapse. These conditions have profoundly influenced the clinical presentation, management, and outcomes of severe infections such as IE, underscoring the need for context-specific evidence from war-affected, resource-limited settings. Therefore, the aim of this study was to identify independent predictors of in-hospital mortality among patients hospitalized with IE at Yemen’s largest tertiary referral center, with particular emphasis on clinically actionable factors relevant to resource-limited, war-affected healthcare settings.

## 2. Patients and Methods

### 2.1 Study Design and Setting

This study employed a prospective, observational cohort design with in-hospital follow-up and was conducted at Al-Thawra Modern General Hospital (TMGH) in Sana’a, Yemen. As the largest public tertiary referral center in the country, TMGH serves as the national hub for advanced medical care, with dedicated cardiology and cardiothoracic surgery units. It admits a diverse spectrum of patients from across Yemen, including critically ill and complex cases, as well as referrals from both public and private health facilities. These characteristics make it a representative setting for investigating the patterns of presentation and short-term outcomes of IE.

The study was approved by the Research Ethics Committee (REC) at Al-Razi University (approval number: Ref: RU/17/FMHS/2023, dated: 20 August 2023) and conducted in accordance with the 2013 Declaration of Helsinki. Administrative permission to access hospital facilities and patient records was obtained from the TMGH Research and Ethics Committee. Written informed consent was obtained from all participants, who were informed of their right to withdraw at any time. Patient confidentiality was ensured by assigning unique identification numbers, de-identifying all personal and health information, and restricting data access to the research team.

### 2.2 Patient Enrollment and Eligibility Criteria

The study population included patients admitted to the cardiology department at TMGH between October 2023 and August 2025 with a diagnosis of IE. Eligibility was established at the time of admission or during hospitalization. This was based on the modified Duke criteria and included both definite and possible cases of IE. Patients with incomplete medical records, those with non-infective forms of endocarditis (e.g., Libman-Sacks endocarditis), or individuals who declined to provide informed consent were excluded.

A total of 60 consecutive patients meeting the inclusion criteria were enrolled during the study period. A consecutive sampling approach was applied to include all eligible patients admitted within the predefined timeframe, ensuring comprehensive coverage of IE cases managed at the hospital.

### 2.3 Data Collection and Clinical Assessment

Data were systematically collected using a structured approach that combined direct patient interviews, review of medical records, and retrieval of diagnostic test results. Within the first 24 hours of admission, a predesigned questionnaire was used to record demographic information, clinical history, type of endocarditis (native valve, prosthetic valve, or device-associated), comorbidities, temperature course, prior administration of antibiotics, laboratory results, and imaging studies. Device-related IE was rare in this cohort, with only one case involving a pacemaker. This case was grouped with PVE for analytical purposes. Laboratory investigations included complete blood counts, renal function tests, urinalysis, and blood cultures. These were performed in the hospital’s central laboratory according to standard protocols. Three sets of blood cultures were obtained at intervals of at least one hour within 24 hours of admission. Echocardiographic findings were documented for all patients, with transthoracic echocardiography (TTE) performed routinely, and transesophageal echocardiography (TEE) conducted when clinically indicated. Additional imaging modalities were retrieved from patient records as needed, including abdominal ultrasound for suspected renal or splenic infarction or abscess, chest radiography, and brain computed tomography for neurological complications. Short-term in-hospital outcomes were prospectively recorded and monitored throughout hospitalization. These included mortality, AHF, septic shock, uncontrolled infection or periannular complications (e.g., valve destruction, abscess formation), embolic events (e.g., ischemic or hemorrhagic stroke, mycotic aneurysm, organ infarctions), empirical treatment, the need for surgical intervention, surgical indications, time to surgery, and causes of death. Data were collected and verified by trained physicians using a standardized case report form to ensure completeness, minimize variability, and reduce information bias.

The primary endpoint was in-hospital mortality. Patients were followed prospectively from admission until hospital discharge or death.

### 2.4 Diagnostic Criteria, Clinical Definitions, and Outcome Measures

IE was diagnosed according to the modified Duke criteria, requiring either two major criteria, one major plus three minor criteria, or five minor criteria. Patients were classified as definite, possible, or rejected cases [13]. Only definite and possible cases were included in the analysis, considering the first episode for patients with recurrent IE.

Acute IE was defined as a rapidly progressive infection with sudden onset of severe symptoms (typically ≤14 days), including high fever and evidence of rapid valvular destruction [12]. Due to limitations in retrospective documentation of exact symptom duration, a specific cutoff time was not applied; instead, the clinical impression of the treating physician at admission was used to classify patients as having acute versus subacute/chronic presentation.

Microbiological confirmation referred to positive blood culture results identifying the causative organism, while culture-negative cases were defined as patients fulfilling the modified Duke criteria without microbiological growth. Prior antibiotic use was defined as the administration of any systemic antibiotic within the two weeks preceding hospital admission.

Echocardiographic evidence was defined as the presence of vegetations, abscesses, new valvular regurgitation, or prosthetic valve involvement on TTE or TEE.

In-hospital outcomes were defined as in-hospital events during the same admission, including cardiac complications (e.g., AHF, conduction abnormalities, myocardial infarction), non-cardiac complications (e.g., AKI, embolic events, sepsis), requirement for surgical intervention, and all-cause mortality.

Septic shock was defined as “a subset of sepsis in which particularly profound circulatory, cellular, and metabolic abnormalities are associated with a greater risk of mortality than with sepsis alone”. It is identified clinically as “the need for vasopressors to maintain a mean arterial pressure of 65 mmHg and a serum lactate level >2 mmol/L (>18 mg/dL) despite adequate fluid resuscitation” [12].

According to the KDIGO (Kidney Disease: Improving Global Outcomes) criteria, AKI was defined as an increase in serum creatinine of at least 26.5 µmol/L (0.3 mg/dL) within 48 hours, or a rise to 1.5 times or more above baseline occurring within 7 days, and/or a reduction in urine output to <0.5 mL/kg/h for a minimum duration of 6 hours [20].

AHF was defined as the rapid onset or worsening of HF symptoms and signs, characterized by pulmonary edema, respiratory distress, elevated jugular venous pressure, new or worsening rales, and/or radiological evidence of acute pulmonary congestion, requiring urgent medical therapy [21].

Uncontrolled infection was defined as persistent clinical and/or laboratory evidence of infection despite appropriate antimicrobial therapy, including sustained fever, hemodynamic instability, persistently elevated inflammatory markers, or microbiological evidence of ongoing infection, often associated with abscess formation, septic emboli, or progressive valvular destruction [22].

Vegetation was defined as a discrete mass of platelets, fibrin, microorganisms, and inflammatory cells attached to the endocardial surface, most commonly on heart valves, as detected by TTE or TEE. Vegetation was considered present if echocardiography revealed a mobile or sessile echogenic structure with irregular borders, consistent with IE, in accordance with the modified Duke criteria [23] and ESC 2015 guidelines [22].

### 2.5 Therapeutic Management: Antibiotic Therapy and Surgical Intervention

All patients received empirical or targeted antibiotic therapy in accordance with hospital protocols and international guidelines for IE. Empirical therapy was initiated upon admission for patients with suspected IE, typically using ceftriaxone (2 g IV twice daily), a penicillin regimen (5 million units IV every 6 hours), or vancomycin (30 mg/kg IV in two doses daily) combined with gentamicin (3 mg/kg single dose daily) during the first two weeks. Critically ill patients or those presenting with acute IE received vancomycin in addition. Therapy was subsequently adjusted based on culture results and antimicrobial susceptibility. Culture-positive cases received pathogen-specific treatment, whereas culture-negative cases were managed with broad-spectrum regimens (vancomycin + cefepime [or antipseudomonal coverage according to epidemiology]). The second phase generally involved penicillin or ceftriaxone monotherapy for an additional two weeks, completing a total 28-day course in patients with native valve IE and extended up to 6 weeks in patients with prosthetic valve IE, with dose adjustments according to renal function when necessary. The choice of antibiotics, route of administration, and treatment duration were documented, and therapy was tailored according to IE type (native versus prosthetic valve) and any complications.

Indications for surgical intervention were determined based on current international guidelines and individual patient clinical status. Surgery was considered in patients with HF secondary to valvular dysfunction, uncontrolled infection despite appropriate antibiotic therapy, presence of large vegetations at high risk of embolization, or complications such as abscess formation or prosthetic valve involvement. The decision to operate was made by the attending cardiology and cardiothoracic surgery teams in consultation with the patient and/or their family. The type of surgical procedure, timing relative to diagnosis, and perioperative outcomes were recorded.

### 2.6 Statistical Analyses

The completeness and accuracy of the data following collection were verified through manual inspection. The dataset was cleaned, coded, and entered into Microsoft Excel before being imported into R software (version 4.2.0; R Foundation for Statistical Computing, Vienna, Austria) for all subsequent analyses. Initially, descriptive statistics were generated to characterize the study population. Categorical variables were summarized as frequencies and percentages. Continuous variables were assessed for normality; normally distributed variables were presented as means ± standard deviations (SD), whereas skewed variables were reported as medians with interquartile ranges (IQR). Univariate analyses were initially performed to explore crude associations between candidate variables and in-hospital mortality. Univariable logistic regression was used where feasible, and crude odds ratios (CORs) with 95% confidence intervals (CIs) were reported. When univariable logistic regression was not applicable due to zero cell counts or complete separation, resulting in non-estimable or infinite odds ratios, categorical variables were compared between survivors and non-survivors using the Chi-square test or Fisher’s exact test, as appropriate. Variables demonstrating an association at *p* < 0.20 were carried forward as candidate variables. These included gender, region, previous IE, IE status, prior stroke, valve type, side of infection, surgical recommendation, and in-hospital complications such as AKI, embolic events, AHF, and uncontrolled infection. Given the small sample size and limited number of outcome events (17 deaths), traditional logistic regression was deemed inappropriate due to risks of overfitting and small-sample bias, especially in the presence of data separation. To address this, a two-step penalized modeling strategy was implemented. First, LASSO regression was applied to the candidate variables to perform data-driven variable selection. LASSO identified three predictors with non-zero coefficients: septic shock, AKI, and uncontrolled infection. Second, the selected predictors were entered into a Firth’s penalized logistic regression model to obtain bias-reduced and stable parameter estimates [24, 25, 26]. With 17 outcome events, the resulting three-predictor model met acceptable event-per-variable criteria for penalized methods. A two-sided *p*-value < 0.05 was considered statistically significant throughout.

## 3. Results 

Of the 60 patients included in this study, 17 died during hospitalization, yielding an in-hospital mortality rate of 28.3%.

### 3.1 Baseline Clinical Characteristics

The baseline demographic and clinical features of the cohort are presented in Table 1. Comparison of continuous variables between survivors and non-survivors using independent *t*-tests (for normally distributed variables) and Mann-Whitney U tests (for skewed variables) revealed no significant differences in age (*p* = 0.834), white blood cells (WBC) count (*p* = 0.242), hemoglobin (HB) level (*p* = 0.479), or serum creatinine (*p* = 0.726). Similarly, Chi-square or Fisher’s exact tests found no significant associations for categorical variables including gender (*p* = 0.066), hypertension (HTN) (*p* = 0.844), diabetes mellitus (DM) (*p* = 0.510), chronic kidney disease (CKD) (*p* = 0.510), prior antibiotic use (*p* = 0.730), blood culture positivity (*p* = 0.397), and type of organism (*p* = 0.574). Among the variables assessed, only urban residence (Chi-square test, *p* = 0.027) and prior stroke (Fisher’s exact test, *p* = 0.041) demonstrated statistically significant associations with in-hospital mortality in univariate analysis.

**Table 1.** **Baseline demographic and clinical characteristics of the study population and their univariate association with in-hospital mortality (n = 60)**.

Variables	Category	Overall	Survival status		Univariate analysis	*p*
			Alive (Number [n] = 43)	Deceased (n = 17)	Crude odds ratio (COR) (95% confidence intervals [CIs])	
Age in years, Mean ± standard deviation (SD)		34.1 ± 17.7	33.7 ± 18.4	34.8 ± 16.3	1.003 (0.972–1.036)	0.834
Gender, n (%)	Male	36 (60.0)	29 (67.4)	7 (41.2)	Reference	0.066
	Female	24 (40.0)	14 (32.6)	10 (58.8)	2.959 (0.930–9.416)	
Region, n (%)	Urban	25 (41.7)	14 (32.6)	11 (64.7)	3.798 (1.165–12.379)	0.027
	Rural	35 (58.3)	29 (67.4)	6 (35.3)	Reference	
Previous infective endocarditis (IE), n (%)	Yes	3 (5.0)	1 (2.3)	2 (11.8)	5.600 (0.473–66.324)	0.172
	No	57 (95.0	42 (97.7)	15 (88.2)	Reference	
Hypertension (HTN), n (%)	Yes	3 (5.0)	2 (4.7)	1 (5.9)	1.281 (0.106–15.132)	0.844
	No	57 (95.0)	41 (95.3)	16 (94.1)	Reference	
Diabetes mellitus, n (%)	Yes	2 (3.3)	2 (4.7)	0 (0.0)	Not applicable (NA)	0.510^#^
	No	58 (96.7)	41 (95.3)	17 (100.0)		
Chronic kidney disease, n (%)	Yes	2 (3.3)	2 (4.7)	0 (0.0)	NA	0.510^#^
	No	58 (96.7)	41 (95.3)	17 (100.0)		
Prior stroke, n (%)	Yes	11 (18.3)	5 (11.6)	6 (35.3)	4.145 (1.060–16.206)	0.041
	No	49 (81.7)	38 (88.4)	11 (64.7)	Reference	
Prior antibiotic use (<2 weeks before admission), n (%)	Yes	44 (73.3)	31 (72.1)	13 (76.5)	1.258 (0.342–4.634)	0.730
	No	16 (26.7)	12 (27.9)	4 (23.5)	Reference	
White blood cells (WBC) (×10^9^/L), Mean ± SD		11.7 ± 7.1	11.0 ± 5.8	13.6 ± 9.7	1.047 (0.970–1.13)	0.242
Hemoglobin (g/dL), Median (interquartile ranges [IQR])		10.0 (9.0, 11.6)	10.0 (8.4, 11.7)	11.0 (9.5, 11.6)	1.095 (0.851–1.408)	0.479
Serum Creatinine (mg/dL, Median (IQR)		0.9 (0.6, 1.2)	1.2 (0.6, 1.2)	1.0 (0.8, 1.25)	0.911 (0.541–1.534)	0.726
Result of blood culture, n (%)	Positive	19 (31.7)	15 (34.9)	4 (23.5)	0.575 (0.159–2.074)	0.397
	Negative	41 (68.3)	28 (65.1)	13 (76.5)	Reference	
Type of organism in culture-positive patients (n = 19; alive = 15, deceased = 4), n (%)	Staphylococcus	8 (42.1)	6 (40.0)^*^	2 (50.0)^*^	1.500 (0.164–13.749)	0.574
	Others	11 (57.9)	9 (60.0)^*^	2 (50.0)^*^	Reference	

**Abbreviations**: CIs, confidence intervals; COR, crude odds ratio; DM, diabetes mellitus; IE, infective endocarditis; HTN, hypertension; IQR, interquartile range; NA, not applicable; SD, standard deviation; WBC, white blood cell. ^*^Percentages are calculated among patients with available culture results. ^#^Chi-square or Fisher’s exact test was used.

### 3.2 Echocardiographic and Disease-Related Characteristics 

Echocardiographic and IE-related characteristics are summarized in Table 2. None of the other echocardiographic variables showed significant differences between survivors and non-survivors upon Chi-square or Fisher’s exact testing: rhythm (*p* = 0.375), vegetation size ≥10 mm (*p* = 0.308), vegetation mobility (*p* = 0.637), vegetation site (*p* = 0.653), side of infection (*p* = 0.152), predisposing heart disease (*p* = 0.361), rheumatic heart disease (RHD) (*p* = 0.583), or severity of pulmonary HTN (*p* = 0.252). Left ventricular ejection fraction (LVEF), assessed by the Mann-Whitney U test, also did not differ significantly between groups (*p* = 0.281). However, patients with prosthetic/device IE had a significantly higher mortality risk compared to those with native valve IE (Fisher’s exact test, *p* = 0.006), with a COR of 11.182 (95% CIs: 1.976–63.273).

**Table 2.** **Echocardiographic findings and univariate association with in-hospital mortality (n = 60)**.

Echo	Category	Overall, n (%)	Survival status, n (%)		Univariate analysis	*p*
			Alive (n = 43)	Deceased (n = 17)	COR (95% CIs)	
Rhythm, n (%)	Sinus	50 (83.3)	37 (86.0)	13 (76.5)	Reference	0.375
	Atrial fibrillation	10 (16.7)	6 (14.0)	4 (23.5)	1.897 (0.461–7.804)	
Left ventricular ejection fraction, Median (IQR)		61.0 (58, 65)	61.0 (58.0, 64.0)	61.0 (59.0, 66.5)	1.050 (0.961–1.149)	0.281
Vegetation present, n (%)	Yes	59 (98.3)	42 (97.7)	17 (100.0)	NA	0.717^#^
	No	1 (1.7)	1 (2.3)	0 (0.0)		
Vegetation size in mm (n = 59; alive n = 42), n (%)	<10 mm	27 (45.8)^*^	21 (50.0)^*^	6 (35.3)	0.545 (0.170–1.747)	0.308
	≥10 mm	32 (54.2)^*^	21 (50.0)^*^	11 (64.7)	Reference	
Vegetation mobility (n = 59; alive n = 42), n (%)	Mobile	50 (84.7)^*^	35 (83.3)^*^	15 (88.2)	1.500 (0.278–8.079)	0.637
	Fixed	9 (15.3)^*^	7 (16.7)^*^	2 (11.8)	Reference	
Vegetation site (n = 59; alive n = 42), n (%)	Mitral	32 (54.2)^*^	22 (52.4)	10 (58.8)	1.299 (0.415–4.061)	0.653
	Others	27 (45.8)^*^	20 (47.6)	7 (41.2)	Reference	
Type of valve, n (%)	Native	52 (86.7)	41 (95.3)	11 (64.7)	11.182 (1.976–63.273)	0.006
	Prosthetic/Device IE	8 (13.3)	2 (4.7)	6 (35.3)	Reference	
Side of the IE, n (%)	Left	45 (75.0)	30 (69.8)	15 (88.2)	3.250 (0.648–16.301)	0.152
	Right	15 (25.0)	13 (30.2)	2 (11.8)	Reference	
Predisposing heart disease, n (%)	Yes	57 (95.0)	40 (93.0)	17 (100.0)	NA	0.361^#^
	No	3 (5.0)	3 (7.0)	0 (0.0)		
Rheumatic heart disease (n = 57; alive n = 40), n (%)	Yes	30 (52.6)	22 (55.0)^*^	8 (47.1)	0.727 (0.233–2.269)	0.583
	No	27 (47.4)	18 (45.0)^*^	9 (52.9)	Reference	
Severity of pulmonary HTN, n (%)	Severe	15 (25.0)	9 (20.9)	6 (35.3)	2.061 (0.598–7.097)	0.252
	Non-severe	45 (75.0)	34 (79.1)	11 (64.7)	Reference	

**Abbreviations**: CIs, confidence intervals; COR, crude odds ratio; HTN, hypertension; IQR, interquartile range; IE, infective endocarditis; NA, not applicable. ^*^Percentages were calculated among patients with available data. ^#^Chi-square or Fisher’s exact test was used.

### 3.3 Management and Treatment Characteristics 

Management-related variables are shown in Table 3. Duration of hospitalization did not differ significantly between survivors and non-survivors (*t*-test, *p* = 0.428). Mode of treatment (medical only vs. medical and surgical) showed no significant association with mortality (Chi-square test, *p* = 0.397). Among patients with a surgical indication, the proportion undergoing surgery did not differ significantly between survivors and non-survivors (Fisher’s exact test, *p* = 0.726).

**Table 3.** **Treatment-related characteristics and their univariate association with in-hospital mortality (n = 60)**.

Variables	Category	Overall, n (%)	Survival status, n (%)		Univariate analysis	*p*
			Alive (n = 43)	Deceased (n = 17)	COR (95% CIs)	
Duration of hospitalization in days, mean ± SD		28.1 ± 22.1	29.8 ± 19.1	24.8 ± 28.0	0.988 (0.960–1.017)	0.428
Mode of treatment, n (%)	Medical only	41 (68.3)	28 (65.1)	13 (76.5)	1.741 (0.482–6.288)	0.397
	Medical and surgical	19 (31.7)	15 (34.9)	4 (23.5)	Reference	
Recommended for surgery, n (%)	Yes	55 (91.7)	38 (88.4)	17 (100.0)	NA	0.176^#^
	No	5 (8.3)	5 (11.6)	0 (0.0)		
Surgery performed (n = 55; alive n = 38), n (%)	Yes	18 (32.7)	13 (34.2)^*^	5 (29.4)	0.801 (0.232–2.769)	0.726
	No	37 (67.3)	25 (65.8)^*^	12 (70.6)	Reference	

**Abbreviations**: CIs, confidence intervals; COR, crude odds ratio; NA, not applicable; SD, standard deviation. ^*^Percentages were calculated among patients with available data. ^#^Chi-square or Fisher’s exact test was used.

### 3.4 In-Hospital Complications 

In-hospital complications showed the strongest univariate associations with mortality (Table 4). All in-hospital complications demonstrated statistically significant associations with mortality on Chi-square or Fisher’s exact testing: AHF (*p* = 0.038), AKI (*p* = 0.001), embolic events (*p* = 0.045), uncontrolled infection (*p* < 0.001), and septic shock (*p* < 0.001).

**Table 4.** **In-hospital complications and their univariate association with mortality (n = 60)**.

In-hospital outcomes	Category	Overall, n (%)	Survival status, n (%)		Univariate analysis	*p*
			Alive (n = 43)	Deceased (n = 17)	COR (95% CIs)	
Acute heart failure, n (%)	Yes	40 (66.7)	25 (58.1)	15 (88.2)	5.400 (1.096–26.612)	0.038
	No	20 (33.3)	18 (41.9)	2 (11.8)	Reference	
Acute kidney injury (AKI), n (%)	Yes	19 (31.7)	8 (18.6)	11 (64.7)	8.021 (2.283–28.185)	0.001
	No	41 (68.3)	35 (81.4)	6 (35.3)	Reference	
Embolic events, n (%)	Yes	23 (38.3)	13 (30.2)	10 (58.8)	3.297 (1.029–10.566)	0.045
	No	37 (61.7)	30 (69.8)	7 (41.2)	Reference	
Uncontrolled infection, n (%)	Yes	30 (50.0)	15 (34.9)	15 (88.2)	14.000 (2.818–69.562)	<0.001
	No	30 (50.0)	28 (65.1)	2 (11.8)	Reference	
Septic shock	Yes	10 (16.7)	1 (2.3)	9 (52.9)	47.250 (5.236–426.425)	≤0.001
	No	50 (83.3)	42 (97.7)	8 (47.1)	Reference	
Any complication	Yes	54 (90.0)	37 (86.0)	17 (100)	NA	0.122^#^
	No	6 (10.0)	6 (14.0)	0 (0.0)		

**Abbreviations**: AKI, acute kidney injury; CIs, confidence intervals; COR, crude odds ratio; NA, not applicable. Among patients with septic shock (n = 10), embolic events occurred in 8 (80.0%). ^#^Chi-square or Fisher’s exact test was used.

### 3.5 Predictors of In-Hospital Mortality 

Consistent with the variables retained through the LASSO selection process, the final Firth-penalized logistic regression model identified septic shock (adjusted odds ratio [AOR] 14.441; 95% CIs: 2.242–176.650; *p* = 0.0036) and AKI (AOR 5.286; 95% CIs: 1.226–26.440; *p* = 0.0264) as the most robust and statistically significant independent predictors of in-hospital mortality (Table 5). Although uncontrolled infection was associated with increased odds of death (AOR 3.672), this variable did not reach statistical significance (*p* = 0.112).

**Table 5.** **Independent predictors of in-hospital mortality based on Firth’s penalized logistic regression**.

Predictor	Firth’s penalized logistic regression	
	Adjusted odds ratio (95% CIs)	*p*
Septic shock	14.441 (2.242–176.650)	0.0036
AKI	5.286 (1.226–26.440)	0.026
Uncontrolled infection	3.672 (0.744–23.011)	0.112

**Abbreviations**: AKI, acute kidney injury; CIs, confidence intervals.

## 4. Discussion 

In this cohort of patients with IE admitted to a tertiary referral center in Yemen, septic shock and AKI emerged as the only independent predictors of in-hospital mortality. Both variables reflect advanced systemic involvement and multiorgan dysfunction, underscoring that short-term mortality in this setting is primarily driven by the severity of acute complications rather than baseline demographic or echocardiographic characteristics. Notably, traditional baseline variables did not retain independent prognostic significance after adjustment, highlighting the central role of organ failure and uncontrolled systemic inflammation in determining early outcomes.

In recent decades, the epidemiological profile of IE has undergone a significant shift, driven by evolving patient demographics and the emergence of new risk factors [27]. When compared with international data, important distinctions emerge. Patients in this cohort were considerably younger, with a mean age of just over 34 years, and RHD was the predominant underlying condition. This contrasts sharply with reports from high-income countries, where IE typically affects older individuals with degenerative valve disease, PVE, or device-related infections [5, 11, 15, 28]. Similar trends of younger age and RHD predominance have been described in studies from sub-Saharan Africa, such as Ethiopia and Egypt [7, 29], underscoring the ongoing burden of preventable structural heart disease in these regions. Moreover, regional variations in demographic and clinical profiles should be considered when interpreting and comparing epidemiological data for IE. The male predominance observed in our cohort is also in line with previous studies [6, 19, 30], while the high proportion of patients from rural areas highlights disparities in healthcare access and delayed presentation.

Blood culture positivity is one of the major diagnostic criteria for IE. Nevertheless, failure to isolate the causative microorganism may occur due to infection with nonbacterial or fastidious pathogens, suboptimal sampling or culture techniques, limitations of automated detection systems, or prior administration of antimicrobial agents before obtaining blood cultures [31, 32]. We documented culture-negative IE in 68.3% of patients. This proportion is higher than that reported in Jordan, Qatar, and Egypt [28, 29, 30], but lower than that reported in Ethiopia [7]. Similarly, a previous study from Yemen reported culture-negative IE in 94% of cases [6]. The markedly higher proportion in that study was likely attributable to the fact that all patients had been on antibiotic therapy for at least four days before admission. The notably low rate of microbiological detection in our cohort can be plausibly attributed to two interrelated factors. First, a considerable proportion of patients (73.3%) had received antibiotic therapy before admission. This is a well-known factor in the literature for significantly reducing the diagnostic yield of blood cultures in IE. Second, as a tertiary referral center, our institution receives patients transferred from peripheral healthcare facilities where empiric antibiotic regimens are often initiated to stabilize the patient’s condition before referral. Importantly, data on whether blood cultures were performed at the referring hospitals before transfer were not systematically available. Even when the data was obtained, results rarely accompanied the patient due to fragmented communication between facilities. This lack of coordination further compounds the diagnostic challenge and limits our ability to ascertain the true microbiological profile of IE in this setting. The combination of prior antimicrobial exposure, referral-related delays, and incomplete interfacility communication is likely to explain the disproportionately high prevalence of culture-negative IE observed in our study. Accordingly, these factors should be carefully considered when interpreting our findings and when drawing comparisons with reports from other regions.

The complications of IE can be broadly categorized into three major groups. The first group includes cardiac complications, such as worsening myocardial infarction or HF. The second group involves the deterioration of non-cardiac organ function and pre-existing comorbidities, including AKI, stroke, DM, and chronic obstructive pulmonary disease. The third group encompasses complications requiring intensive care management, such as sepsis, invasive mechanical ventilation, and dialysis [33]. Patients frequently develop these complications as a consequence of delayed diagnosis and late presentation to the hospital [5, 34]. Previous studies have reported that approximately 80% of patients with IE experience at least one of these complications during the disease course [35, 36]. It has also been reported that major complications were observed in 32.4% of patients at admission, rising to approximately 86% during hospitalization. This highlights the progression of disease before presentation, as well as the critical need for close monitoring and early intervention to prevent further deterioration [35]. Our cohort demonstrated an even higher overall complication rate of 90%, which is among the highest reported to date and substantially exceeds that reported in Jordan (65.2%) and Ethiopia (75.9%) [7, 30]. Cases in Yemen frequently present late and also experience delays in receiving surgical intervention, underscoring the critical need for early diagnosis, timely referral, and prompt management to reduce adverse outcomes. Moreover, the markedly low rate of surgical management within our cohort may have further contributed to the high prevalence of complications.

Surgical intervention was indicated in 92% of our cohort, reflecting the advanced clinical stage at presentation. However, only 33% of those with a surgical indication actually underwent surgery. This rate is comparable to that reported in Saudi Arabia (27%) and Egypt (28%) [28, 37], but substantially lower than in Belgium (56%) [38], and markedly lower than the 50% reported previously in Yemen [6]. This gap between indication and intervention is a critical finding; failure to operate on such a high proportion of indicated patients may partially explain the progression to septic shock (17%) and AKI (31.7%) observed in our cohort, as these complications may have been mitigated by timely surgical source control. Among the 55 patients with a surgical indication, only 18 (32.7%) ultimately underwent surgery. No statistically significant association was observed between surgical intervention and in-hospital mortality (COR 0.801; 95% CIs: 0.232–2.769; *p* = 0.726). This finding should be interpreted with caution, given the small sample size and the observational nature of our study. Patients who underwent surgery may have been systematically different from those who did not (e.g., potentially younger, more hemodynamically stable, or with fewer comorbidities), but the ability to adjust for these differences was limited by the small number of outcome events. Furthermore, patients who deteriorated rapidly and died before surgery were inevitably classified in the non-operated group, introducing a survival bias that may underestimate the potential benefit of surgery. The discrepancy between indication and intervention is largely driven by late clinical presentation and systemic delays. According to the 2023 ESC Guidelines, emergency and urgent surgery must be performed within strictly defined timeframes to be effective [12]. In our resource-limited and war-affected setting, patients often arrive from peripheral facilities already hemodynamically unstable, rendering surgery futile by the time of admission. To improve survival, the primary objective must be to reduce the time between surgical indication and intervention through strengthened communication pathways between peripheral hospitals and tertiary centers, facilitating early transfer before the onset of irreversible organ failure.

The in-hospital mortality rate observed in the present study was 28.3%, which exceeds that reported from Saudi Arabia (24.3%), Jordan (13%), and Japan (14.5%) [5, 30, 37]. This highlights the severe challenges in Yemen, where delayed diagnosis, inadequate referral pathways, and limited availability of specialized cardiac surgery centers result in substantially compromised outcomes. Moreover, systemic deficiencies, including a shortage of trained cardiac surgeons, a lack of advanced perioperative care, and the overall fragility of the healthcare system, further diminish the effectiveness of surgical intervention. Addressing these gaps will require urgent investment in infrastructure, as well as the establishment of specialized cardiac centers, the development of targeted training programs for cardiac teams, and the implementation of streamlined referral systems to ensure timely access to life-saving interventions for patients with IE in Yemen.

Our study found an alarmingly high rate of patients complicated by septic shock (17%), which is a sharp increase from the 6% reported previously [6]. While temporal differences in data collection may partially explain this discrepancy, broader contextual factors also appear to have played a major role. The protracted war has significantly constrained the financial capacity of patients to undergo surgical interventions. Given that surgery is central to the management of septic shock, its underutilization, clearly reflected in our findings compared with prior reports, likely contributed to the increased risk of progression to septic shock. These observations underscore how socioeconomic and political determinants of health intersect with clinical outcomes, amplifying the severity of disease in vulnerable populations.

Antimicrobial therapy remains a cornerstone in the management of sepsis and septic shock [39], with early and appropriate administration consistently associated with improved survival rates [40, 41]. In our cohort, septic shock emerged as the strongest independent predictor of in-hospital mortality, consistent with international data [8, 42]. This finding can be attributed to several factors. Mechanistically, septic shock reflects the transition to a systemic inflammatory state characterized by profound circulatory collapse, uncontrolled bacteremia, and multi-organ failure, all of which severely limit survival despite optimal antibiotic therapy. Moreover, patients in septic shock are often poor surgical candidates due to hemodynamic instability and multi-organ dysfunction [42]. These observations are particularly relevant in low-resource settings such as Yemen, where delayed diagnosis, limited access to intensive care, and the restricted availability of timely cardiac surgery exacerbate the lethality of septic shock. The elevated rate of embolic events in the septic shock subgroup (80.0%) compared with the overall rate (38.3%) represents an additional factor compounding the risk of death in critically ill patients. Taken together, our findings emphasize the critical need for early recognition of sepsis, prompt initiation of effective antimicrobial therapy, and timely multidisciplinary intervention to mitigate the devastating impact of septic shock in IE patients.

AKI is a frequent and clinically meaningful complication of IE, largely driven by the profound hemodynamic disturbances that characterize this disease. Such disturbances may arise from the systemic inflammatory response or from cardiogenic complications, most notably congestive HF, which reduces renal blood flow and precipitates prerenal azotemia and continued renal injury [43, 44]. Studies with animal models have demonstrated that AKI can lead to additional structural and functional damage in cardiac tissue [45]. Recent evidence further highlights that baseline renal vulnerability plays a key role, with the baseline glomerular filtration rate (GFR) reported to be the only independent predictor of subsequent AKI development (OR 0.94, *p* = 0.001) [46]. Beyond its incidence, the clinical impact of AKI is substantial, and patients with IE-associated AKI have markedly higher 90-day mortality [47]. In specialized centers, AKI has been linked to advanced age, elevated baseline creatinine, peripheral embolism, and the development of HF [48]. Moreover, the severity of renal impairment, as reflected by GFR loss and AKI network stage, is strongly associated with in-hospital mortality of IE patients, which itself may reach up to 25% during hospitalization [46]. Large-scale analyses confirm these findings, demonstrating that patients with AKI not only incur significantly greater healthcare utilization and costs [49], but also face increased risks of septic shock (AOR 3.78, 95% CIs: 2.97–4.82, *p* < 0.001) [50]. Long-term outcomes are likewise affected, as early AKI has been associated with higher 1-year mortality (OR 1.65, 95% CIs: 1.03–2.64) and progression to CKD (OR 2.23, 95% CIs: 1.30–3.82) [44]. Collectively, these data demonstrate that AKI is a robust, consistent, and independent predictor of in-hospital mortality in patients with IE. This aligns with our study findings, underscoring the central role of multiorgan dysfunction, particularly renal involvement, in driving early adverse outcomes in this setting.

## 5. Study Limitations

The present study has several limitations that should be considered when interpreting the findings. First, the investigation was conducted at a single tertiary referral center, which may limit the generalizability of our findings, particularly given the high proportion of critically ill cases managed at this institution. Second, the relatively small sample size (n = 60) and limited number of deaths (n = 17) restricted the statistical power and necessitated the use of penalized logistic regression to avoid overfitting. Although this approach improves model stability, it does not fully eliminate the constraints imposed by low event counts. Third, some clinically relevant variables demonstrated substantial imbalance or near-zero variance, such as vegetation presence (98.3%), heart rhythm, and recommended surgical intervention, thus limiting their ability to meaningfully discriminate outcomes despite their potential prognostic relevance. Fourth, although important echocardiographic features (e.g., vegetation characteristics, LVEF, side of infection) did not show significant associations with mortality in our cohort, this may reflect limited statistical power rather than a true absence of effect. Fifth, while blood culture results were obtained systematically, the high proportion of culture-negative cases inherent to resource-limited settings may have reduced the ability to explore organism-specific risks. Sixth, treatment pathways were influenced by real-world constraints, as only one-third of patients underwent surgery despite almost universal surgical recommendations. This disparity, which reflects challenges in timing, access, and feasibility, may have introduced confounding that could not be fully adjusted for. Finally, as all outcomes were restricted to the in-hospital period, long-term morbidity and mortality were not assessed. Future multicenter studies with larger sample sizes and extended follow-up are needed to validate these findings and refine risk prediction in patients with IE.

## 6. Conclusions 

In-hospital mortality was alarmingly high in this cohort of IE patients managed at Yemen’s largest tertiary referral center, underscoring the severity of disease presentation in a resource-limited setting. Following rigorous variable selection and bias-reduced modeling, septic shock and AKI emerged as the most robust and independent predictors of in-hospital mortality, highlighting their critical prognostic significance. These findings highlight the central role of early recognition and aggressive management of systemic complications in improving short-term outcomes of IE, particularly in low-resource environments where advanced diagnostic and therapeutic options may be limited. Future multicenter studies with larger cohorts are warranted to validate these predictors and to refine risk stratification strategies that prioritize the prevention and timely management of life-threatening complications.

## Availability of Data and Materials

The data that support the findings of this study are available from the first author and the corresponding author upon reasonable request.
